# Anti-Cancerous Effect of *Inonotus taiwanensis* Polysaccharide Extract on Human Acute Monocytic Leukemia Cells through ROS-Independent Intrinsic Mitochondrial Pathway

**DOI:** 10.3390/ijms19020393

**Published:** 2018-01-29

**Authors:** Tsai-Ling Chao, Ting-Yin Wang, Chin-Huei Lee, Shuenn-Jiun Yiin, Chun-Te Ho, Sheng-Hua Wu, Huey-Ling You, Chi-Liang Chern

**Affiliations:** 1 Departments of Laboratory Medicine, Kaohsiung Chang Gung Memorial Hospital, Kaohsiung 833, Taiwan; tsaeling@cgmh.org.tw (T.-L.C.); youhling@cgmh.org.tw (H.-L.Y.); 2 Department of Medical Laboratory Science and Biotechnology, Fooyin University, Kaohsiung 831, Taiwan; 3 Department of Laboratory Medicine, Yunlin Christian Hospital, Yunlin 648, Taiwan; phoebe82825@gmail.com; 4 Department of Biological Sciences, National Sun Yat-Sen University, Kaohsiung 804, Taiwan; 5MT970038@live.fy.edu.tw; 5 Department of Nursing, Tajen University, Pintung 907, Taiwan; yiinsj@tajen.edu.tw; 6 Graduate Institute of Medical Sciences, College of Medicine, Taipei Medical University, Taipei 110, Taiwan; homenjer@gmail.com; 7 Department of Biology, National Museum of Natural Science, Taichung 404, Taiwan; shwu@mail.nmns.edu.tw

**Keywords:** *Inonotus taiwanensis*, human acute monocytic leukemia cell line, apoptosis, endonuclease G

## Abstract

Acute leukemia is one of the commonly diagnosed neoplasms and causes human death. However, the treatment for acute leukemia is not yet satisfactory. Studies have shown that mushroom-derived polysaccharides display low toxicity and have been used clinically for cancer therapy. Therefore, we set out to evaluate the anti-cancerous efficacy of a water-soluble polysaccharide extract from *Inonotus taiwanensis* (WSPIS) on human acute monocytic leukemia THP-1 and U937 cell lines in vitro. Under our experimental conditions, WSPIS elicited dose-dependent growth retardation and induced apoptotic cell death. Further analysis showed that WSPIS-induced apoptosis was associated with a mitochondrial apoptotic pathway, such as the disruption of mitochondrial membrane potential (MMP), followed by the activation of caspase-9, caspase-3, and PARP (poly(ADP-ribose) polymerase) cleavage. However, a broad caspase inhibitor, Z-VAD.fmk, could not prevent WSPIS-induced apoptosis. These data imply that mechanism(s) other than caspase might be involved. Thus, the involvement of endonuclease G (endoG), a mediator arbitrating caspase-independent oligonucleosomal DNA fragmentation, was examined. Western blotting demonstrated that WSPIS could elicit nuclear translocation of endoG. MMP disruption after WSPIS treatment was accompanied by intracellular reactive oxygen species (ROS) generation. However, pretreatment with *N*-acetyl-l-cysteine (NAC) could not attenuate WSPIS-induced apoptosis. In addition, our data also show that WSPIS could inhibit autophagy. Activation of autophagy by rapamycin decreased WSPIS-induced apoptosis and cell death. Taken together, our findings suggest that cell cycle arrest, endonuclease G-mediated apoptosis, and autophagy inhibition contribute to the anti-cancerous effect of WSPIS on human acute monocytic leukemia cells.

## 1. Introduction

Acute myeloid leukemia (AML) accounts for about 85% of acute leukemia cases and occurs mostly in young adults [1]. Although chemotherapy is often effective in short-term therapy, drug resistance and relapse are still prevalent. At the same time, numerous chemotherapeutic agents also induce undesirable side effects and increase the risk of developing therapy-related cancer [2]. Thus, developing novel therapies with fewer side effects and extending the overall survival of patients with AML has become urgent.

Recent studies have suggested that chemotherapeutic agents used in cancer therapy are mostly based on mitochondrial apoptosis-inducing effects on cancer cells [3]. Mitochondria-dependent apoptosis is mediated by at least two different pathways. The major pathway, the classic apoptotic pathway, involves the activation of executor caspase-3. A second pathway involves nuclear translocation of mitochondrial proteins such as apoptosis-inducing factor (AIF) and endonuclease G (endoG), which can induce large-scale and oligonucleosomal DNA fragmentation, respectively, independently of caspase recruitment [4,5].

Polysaccharides derived from medicinal mushrooms such as *Lentinus edodes* (Berk.), *Panax ginseng*, *Coriolus versicolor*, and *Agaricus blazei* display very low toxicity compared with chemotherapeutic drugs, and have been used clinically to inhibit tumor growth [6,7,8,9]. Besides the immune response modifiers on the inhibition of tumor growth, recent studies on various tumor cell lines have shown that polysaccharides can have direct cytotoxic effects [10]. Therefore, developing various kinds of mushroom-derived polysaccharides might be a promising strategy for the treatment of cancer.

*Inonotus taiwanensis* is a new species of basidiomycetous fungus inhabiting *Trema orientalis* [11]. We prepared a water-soluble polysaccharide extract (WSPIS) from the fruiting bodies of *Inonotus taiwanensis* and explored the underlying cytotoxic mechanisms of WSPIS using human acute monocytic leukemia cell lines. To the best of our knowledge, this is the first report studying the anti-cancerous effect of WSPIS. In this study, we found that cell cycle disturbance, mitochondria-mediated apoptosis, and autophagy inhibition contributed to the growth-inhibitory effects of WSPIS.

## 2. Results

### 2.1. Anti-Proliferative Activity of WSPIS in Human Monocytic Leukemia Cells

**Figure 1:**
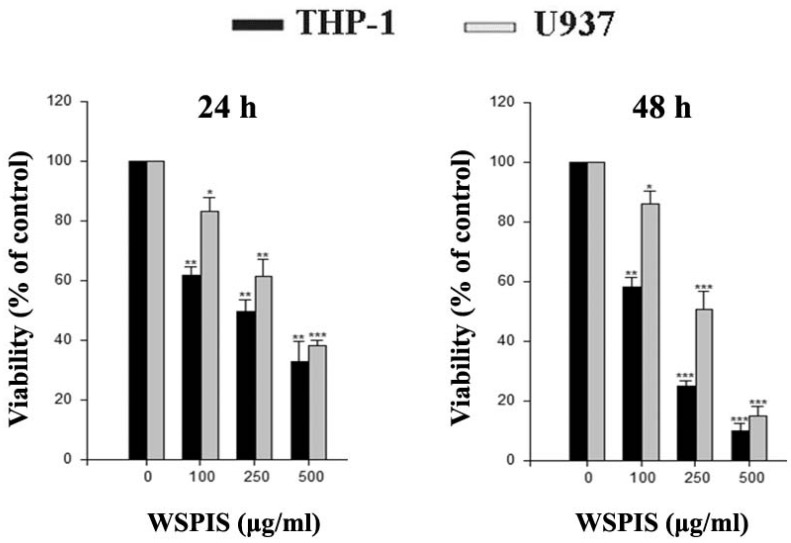
Cytotoxic effect of a water-soluble polysaccharide extract from *Inonotus taiwanensis* (WSPIS) on human monocytic leukemia cell lines. THP-1 and U937 leukemia cells were treated with WSPIS (100, 250, and 500 µg/mL) for 24 and 48 h. Cell viability was determined by the trypan blue exclusion assay. The results are expressed as mean ± SD of three independent experiments. *, ** and *** denote the data that are significantly different from control at *p * < 0.05, *p* < 0.01 and *p* < 0.001, respectively.

To investigate the cytotoxic effect of WSPIS, THP-1 and U937 cells were exposed to various concentrations of WSPIS for 24 and 48 h, and the extent of cell death was assessed by trypan blue staining. As shown in Figure 1, WSPIS induced dose-dependent cytotoxicity in these cells with IC_50_ values of about 137 μg/mL and 255 μg/mL for THP-1 and U937 cells, respectively, at 48 h.

### 2.2. WSPIS Caused DNA Damage and Delayed Cell Cycle Progression

**Figure 2:**
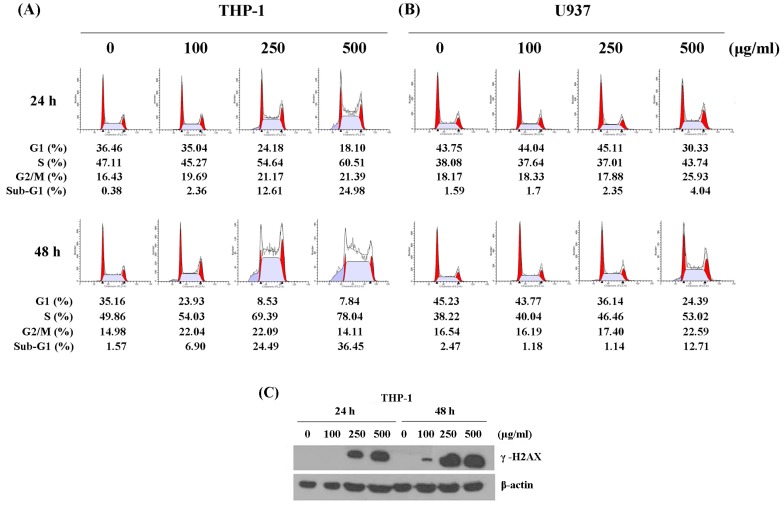
WSPIS disturbed cell cycle progression and caused DNA damage. (**A**,**B**) DNA content analysis. THP-1 and U937 cells were treated with various doses of WSPIS (100, 250 and 500 µg/mL) for 24 and 48 h. DNA content was analyzed using flow cytometry after propidium iodide staining. (**C**) The protein level of γ-H2AX was determined by Western blotting in WSPIS-treated THP-1 cells. DNA content histograms and Western blotting data are representative of three independent experiments.

To determine whether the anti-proliferative effect of WSPIS was due to delayed cell cycle progression, we performed cell cycle analyses. As shown in Figure 2A,B, WSPIS caused a progressive increase in the percentage of cells in the S phase in THP-1 and U937 cells. Whether the cell cycle disturbance following WSPIS treatment was due to DNA damage, the protein level of γ-H2AX, a sensitive marker of DNA double-strand breaks [12], was determined by Western blotting. As shown in Figure 2C, WSPIS increased the protein level of γ-H2AX in a dose- and time-dependent manner in THP-1 cells. These results imply that the cell cycle arrest induced by WSPIS at the S phase was due to the effect of DNA damage.

### 2.3. WSPIS Induced Apoptosis in THP-1 Cells

**Figure 3:**
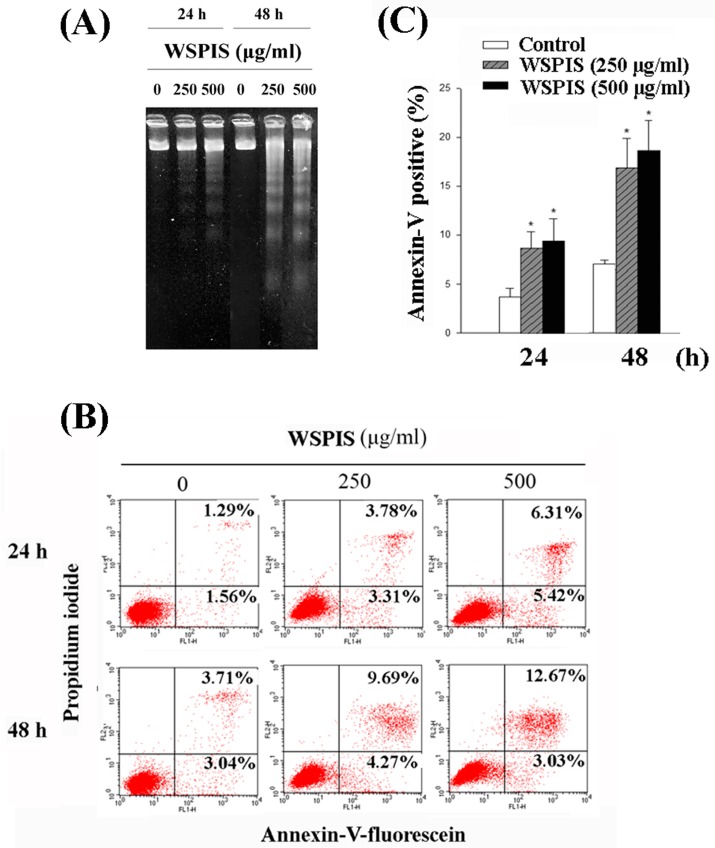
Apoptotic effects of WSPIS in THP-1 cells. Cells were treated with various concentrations of WSPIS (250 and 500 µg/mL) for 24 and 48 h. (**A**) DNA fragmentation was analyzed by agarose gel electrophoresis. Data are representative of three independent experiments. (**B**) Annexin-V-positive cells were analyzed by flow cytometry after annexin-V/propidium iodide staining. (**C**) Apoptotic cells (annexin-V-positive) are expressed as mean ± SD of three independent experiments. * denotes the data that are significantly different from control at *p* < 0.05.

Cell cycle analysis also revealed that WSPIS increased the percentage of cells in the sub-G1 fraction in both THP-1 and U937 cells (Figure 2A,B). To further demonstrate that the demise of these cells in the sub-G1 fraction was apoptotic in nature, we performed agarose gel electrophoresis and annexin-V/propidium iodide (PI) staining assays in WSPIS-treated THP-1 cells. As shown in Figure 3A, oligonucleosomal DNA fragmentation appeared in WSPIS-treated THP-1 cells. The annexin-V/PI staining data also reveal that WSPIS increased the population of apoptotic cells (annexin-V positive) at 48 h from 6.75% of control cells to 13.96% and 15.7% of cells treated with 250 and 500 µg/mL WSPIS, respectively (Figure 3B,C).

### 2.4. WSPIS Induced Reactive Oxygen Species (ROS) Production and Activated Mitochondrial Apoptotic Pathway

**Figure 4:**
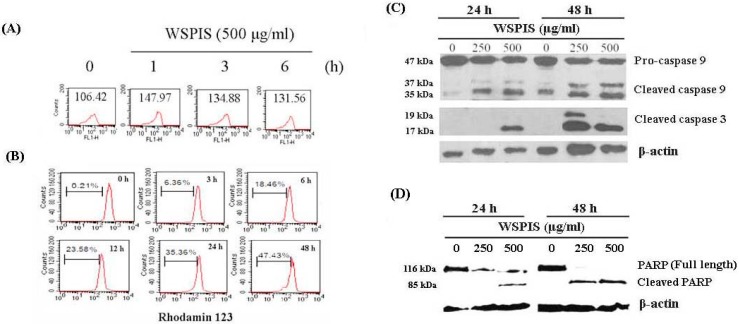
WSPIS induced the mitochondrial apoptotic pathway in THP-1 cells. (**A**) Effect of WSPIS on reactive oxygen species (ROS) production. Cells were treated with 500 μg/mL WSPIS for 1, 3, and 6 h. Intracellular ROS was analyzed using flow cytometry after DCF-DA staining. (**B**) Effect of WSPIS on loss of the mitochondrial membrane potential (MMP). Cells were treated with WSPIS (500 μg/mL) and harvested at the indicated times. The MMP was analyzed using flow cytometry after rhodamine 123 staining. (**C**,**D**) Analysis of the cleaved products of caspase-3, caspase-9, and poly(ADP-ribose) polymerase (PARP). Cells were treated with WSPIS (0, 250, and 500 μg/mL) for 24 and 48 h, and the cleaved products of caspase-3, caspase-9, and PARP were detected by Western blotting. Data are representative of three independent experiments.

To determine whether WSPIS-induced apoptosis in THP-1 cells was associated with reactive oxygen species (ROS)-mediated oxidative stress, we measured the intracellular ROS production by 2′,7′-dichlorofluorescin diacetate (DCF-DA) staining. As indicated in Figure 4A, the level of ROS drastically increased at 1 h and decreased with time thereafter. Next, we examined mitochondrial membrane potential (MMP) by rhodamine 123 staining. As shown in Figure 4B, during the 3–48 h time period, WSPIS was capable of inducing a time-dependent increase in the population that lost MMP, from 6.36% to 47.43%. The decreased MMP usually coincided with the release of cytochrome c from the mitochondria into the cytosol, resulting in the activation of caspase-9 and caspase-3. Therefore, we evaluated the activation of these two caspases by Western blotting. As shown in Figure 4C, WSPIS increased the formation of the cleavage products of caspase-3 (17 kDa) and caspase-9 (37 kDa). In addition, cleaved poly(ADP-ribose) polymerase (PARP) (85 kDa), a product of active caspase-3, also increased following WSPIS treatment (Figure 4D). These results imply that the mitochondrial apoptotic pathway was triggered in WSPIS-treated THP-1 cells.

### 2.5. WSPIS Induced Caspase-Independent but EndoG-Mediated Apoptosis

**Figure 5:**
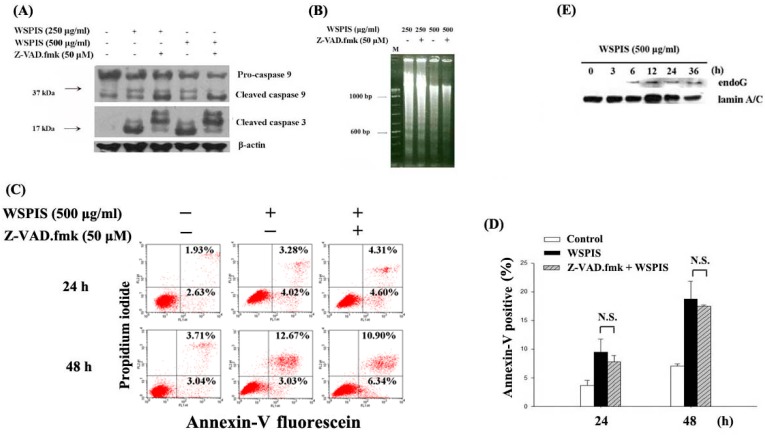
Caspase-independent apoptosis in WSPIS-treated THP-1 cells. (**A**–**D**) Cells were pretreated with or without Z-VAD.fmk (50 μM) for 1 h followed by WSPIS treatment for 24 and 48 h. (**A**) Cleaved products of caspase-3 and caspase-9 (17 and 37 kDa, respectively) were measured by Western blotting. (**B**) Oligonucleosomal DNA fragmentation was determined by agarose gel electrophoresis. M is the DNA markers. (**C**) Apoptotic cells (annexin-V-positive) were analyzed using flow cytometry after annexin-V/propidium iodide staining. (**D**) Apoptotic cells are expressed as mean ± SD of three independent experiments. N.S. = Not significant. (**E**) Nuclear translocation of endoG. Cells were treated with 500 μg/mL WSPIS for the indicated time periods. The levels of nuclear endoG were determined by Western blotting. Results are representative of three independent experiments.

To confirm the role of caspases in WSPIS-activated mitochondrial apoptotic pathway, THP-1 cells were pretreated with the pan-caspase inhibitor, Z-VAD.fmk (50 μM), for 1 h before WSPIS treatment. Western blotting results show that Z-VAD.fmk inhibited cleaved caspase-3 (17 kDa) and cleaved caspase-9 (37 kDa) formation (Figure 5A), implying that Z-VAD.fmk was functional. However, it could not inhibit oligonucleosomal DNA fragmentation and the annexin-V-positive population induced by WSPIS (Figure 5B–D). These findings suggest that WSPIS-induced apoptosis was through a caspase-independent pathway. Endonuclease G (endoG) is a mitochondrial protein that translocates into the nucleus during the caspase-independent apoptotic process, where it mediates oligonucleosomal DNA fragmentation [5]. To investigate the possible role of endoG in WSPIS-induced apoptosis in THP-1 cells, nuclear translocation of endoG was examined by Western blotting. As shown in Figure 5E, increased levels of nuclear endoG were observed at 6 h until 36 h following WSPIS stimulation. These results imply that endoG translocation into the nucleus may have been involved in mediating oligonucleosomal DNA fragmentation in WSPIS-treated THP-1 cells.

### 2.6. WSPIS-Induced Apoptosis through the ROS-Independent Pathway

**Figure 6:**
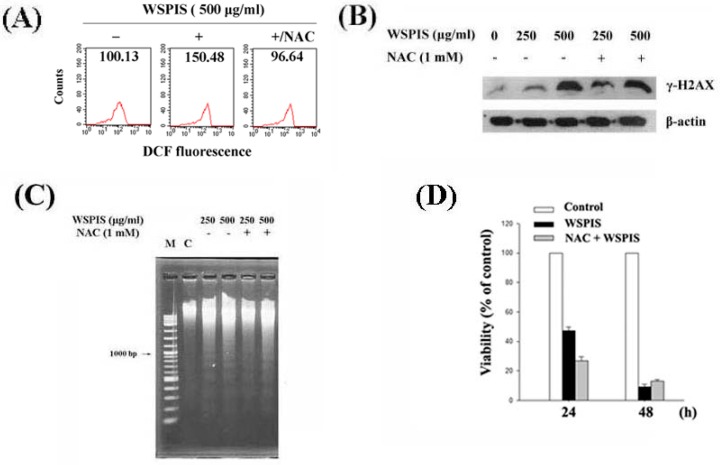
Effect of *N*-acetyl-l-cysteine (NAC) on WSPIS-induced DNA damage and apoptosis in THP-1 cells. Cells were pretreated with NAC (1 mM) for 1 h followed by WSPIS treatment for the indicated time periods. (**A**) Intracellular ROS content was evaluated at 1 h after 500 μg/mL WSPIS treatment by measuring oxidized DCF fluorescence using flow cytometry. (**B**) The protein level of γ-H2AX was determined by Western blotting at 48 h after WSPIS treatment. (**C**) Oligonucleosomal DNA fragmentation was analyzed by agarose gel electrophoresis at 48 h after WSPIS treatment. (**D**) Cell viability was determined by the trypan blue exclusion assay at 24 and 48 h after WSPIS treatment. Values are expressed as a percentage compared to the control. Data are representative of three independent experiments.

To determine the role of ROS in WSPIS-induced γ-H2AX formation and apoptosis in THP-1 cells, we used an antioxidant, *N*-acetyl-l-cysteine (NAC), to suppress intracellular ROS production. As shown in Figure 6A, 1 mM NAC was capable of decreasing ROS production induced by 500 μg/mL WSPIS, as reflected by the decrease in DCF fluorescence from 150.48 to 96.64. However, the protein level of γ-H2AX, oligonucleosomal DNA fragmentation, and cell death were not rescued by NAC (Figure 6B–D). These results suggest that WSPIS-induced DNA damage and apoptosis were through a ROS-independent pathway.

### 2.7. WSPIS Inhibited Autophagy in THP-1 Cells 

**Figure 7:**
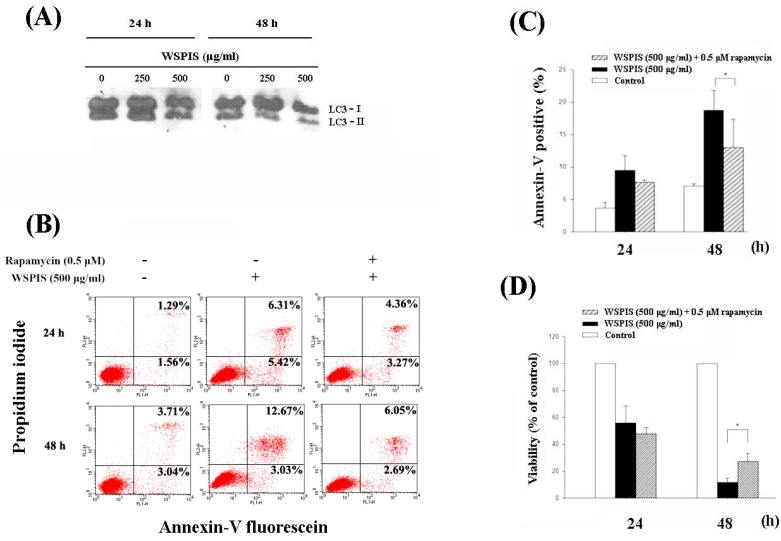
WSPIS inhibited autophagy. (**A**) Cells were treated with various concentrations of WSPIS (250 and 500 μg/mL) for 24 h and 48 h. The protein levels of LC3-I and LC3-II were measured by Western blotting. (**B**) Autophagy induction by rapamycin partially rescued WSPIS-induced apoptosis. THP-1 cells were pretreated with rapamycin (0.5 μM) for 1 h, followed by 500 µg/mL WSPIS treatment for 24 and 48 h. Apoptotic cells were analyzed by using flow cytometry after annexin-V/propidium iodide staining. (**C**,**D**) Rapamycin partially rescued WSPIS-induced apoptosis and cell death after 48 h treatment. Apoptosis and cell viability are expressed as mean ± SD of three independent experiments. Cell viability was determined by trypan blue staining. * denotes the data that are significantly different between WSPIS-only and WSPIS-combined rapamycin groups at *p* < 0.05.

Microtubule-associated protein light chain 3 (LC3) is a widely used marker to monitor autophagy. Upon the induction of autophagy, cytosolic LC3-I is degraded and converts to autophagosome membrane-bound LC3-II. Thus, the autophagic membrane LC3-II reflects the autophagic process [13]. To determine whether autophagic cell death contributed to WSPIS-induced apoptosis and cell death, we first analyzed LC3-II expression by Western blotting. As shown in Figure 7A, WSPIS decreased LC3-II expression in a dose- and time-dependent manner. These results suggest that WSPIS inhibited autophagy. To investigate the role of autophagy in WSPIS-induced apoptosis and cell death, we used the autophagy inducer rapamycin to promote autophagy. As shown in Figure 7B,C, rapamycin partially decreased WSPIS-induced annexin-V-positive cells at 48 h from 15.70% for control to 9.74% for WSPIS treatment. In the same vein, rapamycin also reduced WSPIS-induced cell death (Figure 7D). On the basis of these data, we conclude that WSPIS did not induce THP-1 cells to undergo autophagy. Conversely, activation of autophagy protected THP-1 cells from WSPIS-induced cell death.

## 3. Discussion

Chemotherapy is often the first choice for AML treatment. However, serious side effects and a low overall survival rate due to the development of drug resistance are issues that need to be solved. Therefore, development of novel therapeutic agents with less cytotoxicity has become of interest. In search of novel antitumor agents with much less cytotoxicity, mushroom-derived polysaccharides are becoming more and more attractive due to their low toxicity and safety in clinical uses. In this study, we investigated the anti-cancerous effects of polysaccharide extracts from the fruiting bodies of *Inonotus taiwanensis* on human monocytic leukemia cells.

Our results clearly indicate that WSPIS had remarkable cytotoxic effects in THP-1 and U937 cells. The flow cytometry results show that WSPIS increased the percentage of cells at the S phase and sub-G1 fraction in these two cell lines, but to a lesser extent in U937 cells (Figure 2A,B). For this reason, the signaling pathway related to WSPIS-induced growth retardation was investigated in THP-1 cells. Western blotting data also show that WSPIS increased the protein level of γ-H2AX in THP-1 cells (Figure 2C). These results imply that DNA damage might have resulted in the cell cycle disturbance and the accumulation of the sub-G1 fraction in the WSPIS-treated THP-1 cells.

In addition, we performed agarose gel electrophoresis and annexin-V/propidium iodide staining assays to confirm that the cells in the sub-G1 fraction were apoptotic in nature. As shown in Figure 3A–C, a dose- and time-dependent increase of oligonucleosomal DNA fragmentation and annexin-V-positive apoptotic cells appeared in WSPIS-treated THP-1 cells. Among the apoptotic pathways, the mitochondria-mediated pathway is the key pathway exploited by chemotherapeutic agents [3]. In our study, we found that WSPIS decreased the mitochondrial membrane potential (MMP) and activated caspase-9 and caspase-3 (Figure 4B,C), implying that mitochondria-mediated caspase activation is involved in WSPIS-induced apoptosis. However, a pan-caspase inhibitor such as Z-VAD.fmk could not prevent WSPIS-induced apoptosis (Figure 5B–D). These results imply that WSPIS-induced apoptosis might be through the mitochondria-mediated pathway but a caspase-independent pathway. Endonuclease G (endoG) is a mitochondrial protein arbitrating caspase-independent apoptosis. Therefore, we focused on the endoG pathway. As shown in Figure 5E, the increased level of nuclear endoG was first observed 6 h after WSPIS treatment, which coincided with significant MMP collapse. From the above data, we suggest that WSPIS-induced apoptosis might be through the endoG-mediated pathway.

Excessive ROS can cause DNA damage and initiate apoptosis [14,15]. In this study, the time-course experiments showed that the elevation of ROS occurred earlier than mitochondrial membrane potential disruption (Figure 4A,B), suggesting that ROS may be a trigger activating the mitochondria-mediated apoptotic pathway. However, the ROS scavenger NAC neither decreased apoptosis nor reduced the protein level of γ-H2AX in WSPIS-treated THP-1 cells (Figure 6B–D). These results imply that WSPIS-induced apoptosis is through a ROS-independent pathway in THP-1 cells.

Recent studies have shown that autophagy promotes cancer cell survival and inhibition of autophagy improves outcome in cancer therapy [16,17,18]. In this study, we found that WSPIS inhibited autophagy in THP-1 cells (Figure 7A). An autophagy inducer, rapamycin, partially reduced WSPIS-induced apoptosis and cell death (Figure 7B–D). These results suggest that autophagy plays a pro-survival role in WSPIS-treated THP-1 cells.

## 4. Materials and Methods 

### 4.1. Reagents

Rhodamine 123, propidium iodide, and 2′,7′-dichlorodihydrofluorescein diacetate (DCFH-DA) utilized herein were acquired from Molecular Probes (Eugene, OR, USA). Rabbit polyclonal antibodies to cleaved caspase-3 (#9661), caspase-9 (#9502), γ-H2AX (#9718), and endonuclease G (#4969) were purchased from Cell Signaling Technology (Danvers, MA, USA). Antibody for LC3B/MAP1LC3B (#NB600-1384) was from Novus Biologicals (Novus Biologicals, Littleton, CO, USA). Pan-caspase inhibitor (Z-VAD.fmk) and mouse monoclonal antibody specific for β-actin were from Sigma-Aldrich (Saint Louis, MO, USA). Monoclonal anti-poly (ADP-ribose) polymerase (PARP) antibody (#556494) and FITC annexin-V apoptosis detection kit were purchased from BD Pharmingen (San Diego, CA, USA).

### 4.2. WSPIS Polysaccharide Extract Preparation and Total Carbohydrate Measurement

Recently, a new species of fungus, named *Inonotus taiwanensis*, was discovered and deposited in the herbarium of the National Museum of Natural Science, Taiwan (TNM) (11). Dried *Inonotus taiwanesis* fruiting bodies (3 kg) were extracted twice with hot distilled water (*w*/*v*, 1:10) at 100 °C (for 3 h each). After centrifugation, the aqueous extracts were concentrated to 200 mL, and four volumes of 95% ethanol were added. The mixture was kept overnight at 4 °C and centrifuged at 7500× *g* for 15 min to precipitate the polysaccharide. The precipitate was dried in a freeze drier to produce WSPIS (142.3 g, 4.74%). Total carbohydrate content in WSPIS was 50 mg/g WSPIS by phenol-sulfuric acid method [19].

### 4.3. Cell Culture and Treatment with WSPIS

The human THP-1 and U937 cell lines were purchased from the Food Industry Research and Development Institute (Hsin-Chu, Taiwan) and maintained in RPMI-1640 media supplemented with 10% FBS. The cells were grown at 37 °C and 5% CO_2_ in a humidified environment. In addition, cells at a density of 1.5 × 10^5^/mL was used to evaluate the effects of WSPIS in THP-1 cells.

### 4.4. Cell Viability Assay

Cells were grown and treated with WSPIS (250 and 500 μg/mL) for 24 and 48 h. At the end of each experimental period, cells were collected by centrifugation and counted using a hemocytometer. Cell viability was determined by the trypan blue exclusion method.

### 4.5. Flow Cytometry Analysis of DNA Content

Cell cycle distribution and sub-G1 population were evaluated using propidium iodide as described previously [20]. Briefly, cells were treated with different concentrations of WSPIS for 24 and 48 h. Adherent and floating cells were pooled and fixed in a PBS-methanol solution for 16 h at 4°C. Later, cell pellets were suspended in 0.5 mL PBS containing 2.4 μL RNase A (10 μg/mL) and the same volume of propidium iodide (10 μg/mL) in the dark for 30 min. The stained cells were analyzed using a Becton-Dickinson FACS-Calibur flow cytometer (BD Biosciences, San Jose, CA, USA).

### 4.6. Agarose Gel Electrophoresis of DNA Fragmentation

Oligonucleosomal DNA fragmentation was evaluated using agarose gel electrophoresis as described previously [21]. Briefly, cells were treated with WSPIS (250 and 500 μg/mL) for 24 and 48 h. Cell pellets were resuspended in 30 μL of ice-cold Tris-EDTA buffer (pH 8.0), to which were added 12 volumes of 6 M guanidine-HCl, 1 volume of 7.5 M ammonium acetate, 1 volume of 20% sodium dodecyl sulfate, and 1 volume of proteinase K (3 mg/mL). Cell lysates were incubated at 50 °C for 16 h and genomic DNA was extracted using phenol/chloroform/isoamyl alcohol (25:24:1) and precipitated with absolute alcohol. DNA samples were electrophoresed on a 2% agarose gel and visualized with ethidium bromide staining under UV illumination.

### 4.7. Annexin-V/Propidium Iodide (PI) Staining

THP-1 cells were treated with WSPIS (250 and 500 μg/mL) for 24 and 48 h. Apoptosis was determined using annexin V/propidium iodide staining according to the manufacturer’s protocol. In brief, cells (1 × 10^5^) were washed and suspended in 100 μL of annexin-V binding buffer and stained with annexin-V-FITC (5 μL) and propidium iodide (5 μL) in the dark for 10 min at 37 °C. The early and late apoptotic cells were analyzed using a Becton Dickinson FACS-Calibur flow cytometer.

### 4.8. Measurement of Mitochondrial Membrane Potential (MMP) by Flow Cytometry

The MMP was determined by flow cytometry after staining with rhodamine 123. Cells were grown and treated with 500 μg/mL WSPIS for 3, 6, 12, 24, and 48 h. At the end of each experimental period, cells were incubated with rhodamine 123 (5 μM) for 30 min in the dark. The MMP was determined by analyzing the fluorescent level of rhodamine 123 using a Becton Dickinson FACS-Calibur flow cytometer.

### 4.9. Cell Lysate Preparation and Immunoblotting

Total cell and nuclear lysates were prepared as described previously [21]. Briefly, for total cell lysate preparation, control and WSPIS-treated cells were collected by centrifugation and then the pellets were suspended at 4 °C for 1 h in a lysis buffer (50 mM Tris-HCl (pH 8.0), 150 mM NaCl, 1 mM EDTA, 1 mM EGTA, 1% Triton X-100, 1 mM phenylmethylsulfonyl fluoride) supplemented with protease inhibitors. For nuclear lysate preparation, cells were suspended in hypotonic buffer (10 mM HEPES (pH 7.5), 10 mM KCl, 0.1 mM EDTA, 0.1 mM EGTA, 1 mM NaF, 1 mM DTT, 1 mM Na_3_VO_4_, 1 mM phenylmethanesulfonyl fluoride) and on ice for 15 min. Vigorously vortexed after 0.5% Nonidet P-40 was added. The nuclei were pelleted and resuspended in high-salt buffer (20 mM HEPES (pH 7.5), 400 mM NaCl, 1 mM phenylmethanesulfonyl fluoride). The nuclear lysates were centrifuged and the supernatants were frozen at −80 °C. For Western blotting, equal amounts of proteins were resolved on 12% polyacrylamide gel and transferred to a nitrocellulose membrane. The membrane was incubated with the appropriate primary antibodies in blocking buffer. Detection was performed with an enhanced chemiluminescence system and exposed to X-ray films.

### 4.10. Statistics

All experiments were carried out in triplicate. Data are expressed as mean ± SD and analyzed to determine statistical significance of difference between the control and test group by Student’s *t*-test. *p* < 0.05 was considered statistically significant. Levels of statistical significance are indicated by asterisks (* *p* < 0.05, ** *p* < 0.01 or *** *p* < 0.001).

## 5. Conclusions

In conclusion, our findings demonstrate that WSPIS exhibits a growth retardation effect on human monocytic leukemia cell lines in vitro through cell cycle progression delay and induction of endoG-mediated apoptotic cell death. These data suggest that WSPIS can be a candidate agent for the development of novel anticancer drugs against acute leukemia.
